# Identification of Predictive Biomarkers for Lymph Node Involvement in Obese Women With Endometrial Cancer

**DOI:** 10.3389/fonc.2021.695404

**Published:** 2021-07-07

**Authors:** Vanessa M. López-Ozuna, Liron Kogan, Mahmood Y. Hachim, Emad Matanes, Ibrahim Y. Hachim, Cristina Mitric, Lauren Liu Chen Kiow, Susie Lau, Shannon Salvador, Amber Yasmeen, Walter H. Gotlieb

**Affiliations:** ^1^ Division of Gynecologic Oncology, Jewish General Hospital, McGill University, Montreal, QC, Canada; ^2^ Segal Cancer Center, Lady Davis Institute of Medical Research, McGill University, Montreal, QC, Canada; ^3^ Department of Gynecologic Oncology, Hadassah Medical Center, affiliated with Hebrew University Hadassah Medical School, Jerusalem, Israel; ^4^ College of Medicine, Mohammed Bin Rashid University of Medicine and Health Sciences, Dubai, United Arab Emirates; ^5^ College of Medicine, Sharjah Institute for Medical Research, University of Sharjah, Sharjah, United Arab Emirates

**Keywords:** lymph node, molecular markers, endometrial cancer, obesity, tumor biomarkers, body mass index (BMI)

## Abstract

Obesity, an established risk factor for endometrial cancer (EC), is also associated to increased risks of intraoperative and postoperative complications. A reliable tool to identify patients at low risk for lymph node (LN) metastasis may allow minimizing the surgical staging and omit lymphadenectomy in obese patients. To identify molecular biomarkers that could predict LN involvement in obese patients with EC we performed gene expression analysis in 549 EC patients using publicly available transcriptomic datasets. Patients were filtrated according to cancer subtype, weight (>30 kg/m^2^) and LN status. While in the LN+ group, NEB, ANK1, AMIGO2, LZTS1, FKBP5, CHGA, USP32P1, CLIC6, CEMIP, HMCN1 and TNFRSF10C genes were highly expressed; in the LN- group CXCL14, FCN1, EPHX3, DDX11L2, TMEM254, RNF207, LTK, RPL36A, HGAL, B4GALNT4, KLRG1 genes were up-regulated. As a second step, we investigated these genes in our patient cohort of 35 patients (15 LN+ and 20 LN-) and found the same correlation with the in-silico analysis. In addition, immunohistochemical expression was confirmed in the tumor tissue. Altogether, our findings propose a novel panel of genes able to predict LN involvement in obese patients with endometrial cancer.

## Introduction

Endometrial cancer (EC) is considered as the fourth most common malignancy among women and the second leading cause of death from gynecological cancers (1, 2). The standard surgical treatment of EC includes hysterectomy, bilateral salpingo-oophorectomy, and lymph node evaluation, either by sentinel lymph node mapping or by lymphadenectomy (3–6).

Obesity is defined by body mass index >30 kg/m^2^ (7) and is believed to be in part responsible for the increasing incidence of EC in the last 30 years (8, 9). In the United States, around 57% of the EC cases were linked to overweight and obesity (10), and this is associated with the increase of estrogen production in the adipose tissue (11, 12).

Patients suffering from obesity represent a considerable challenge during the intra- and post-operative periods that has been associated with short and long term adverse clinical outcomes (13–17). In a previous report, our team assessed several clinical factors associated with LN involvement in obese patients with EC and found that sentinel node detection (SLN) and lymph node (LN) dissection decreased with increasing BMI, and lymph node involvement was inversely correlated with BMI (18). In view of the lower detection rate of SLN, the decreased risk of LN involvement, and the increased operative risk of LN dissection in this patient population, it is questionable whether LN dissection should be performed if SLN mapping failed in obese patients with EC.

New improvements have been made in genetic and molecular profiling that have facilitated the identification of gene signatures as diagnostic tools for clinical decision making, or as creators of targets that could have an impact in therapeutic approaches (19, 20). Here, we aimed to identify molecular markers, as predictors of LN involvement in obese women with EC.

## Material and Methods

### Study Design

This study was conducted in the division of Gynecologic Oncology at the Segal Cancer Center of the Jewish General Hospital. Between December 2007 and August 2017, 722 patients with uterine cancer underwent surgical staging in our institution. In this study we included patients with histologic diagnosis of endometrioid endometrial carcinoma, BMI above 30kg/m^2^, known LN status after surgery, and material in our biobank for RNA extraction and processing. Patient’s exclusion criteria were: patients with sarcomas, patients who received neoadjuvant therapy, patients whose body mass index was below 30kg/m^2^. All patients underwent robotically assisted surgical staging which included total hysterectomy, bilateral salpingo-oophorectomy and LN assessment by either LN dissection or SLN mapping. Tumor samples were stored in the gynecologic oncology tumor bank for further processing.

### Ethics and Clinical Samples

The study was approved by the Institutional Review Ethics Board (protocol #2019-1547), with annual reviews. Prior to surgery, informed consent was obtained from all participants. Tissue samples were collected at the time of surgery, preserved in RNAlater (Qiagen) and stored at −80°C in the gynecologic oncology tumor bank (protocol #03-041). All samples were selected after histologic confirmation, and high tumor content (>70% tumor content) was confirmed by a gynecologic pathologist. Clinical data for each patient was available.

### Clinical Data Collection

All clinical data was entered in a prospective fashion into a clinical data base. We retrospectively analyzed the perioperative data for all cases. The operative, clinical, pathological and survival data were retrieved from electronic medical records.

### Antibodies

The following antibodies were used to perform the immunohistochemistry: CEMIP (ABclonal #A8587) 1:200 dilution, RPL36A (Novus biologicals #NBP2-38036) 1:25 dilution, CXCL14 (Thermofisher #10468-1-AP) 1:100 dilution, EPHX3 (Thermofisher #PA5-52992) 1:10 dilution, TMEM254 (Novus Biologicals, NBP2-68939) 1:50 dilution, LZTS1 (Novus Biologicals, NBP2-17195) 1:50 dilution, CLIC6 (Novus Biologicals, NBP2-38062) 1:10 dilution, HMNC1 (Thermofisher #PA5-62458) 1:50 dilution.

### RNA Extraction and Reverse Transcriptase Real-Time PCR

Total RNA was extracted from Formalin-Fixed, Paraffin-Embedded tissue (FFPE) using PureLink RNA Mini Kit (Thermo Fisher). First strand cDNA was synthesized using 5X All-In-On MasterMix (MasterMix-LR, Diamed) per manufacturer’s protocol. Reverse transcriptase real-time PCR (RT-PCR) was carried out on 96-well plates using iTaq Universal SYBR Green Supermix (BioRad). Concentrations for each sample were measured using the NanoDrop ND-100 spectrophotometer 119 (NanoDrop Technologies, Wilmington, DE, USA) and Qubit (Thermo Fischer Scientific, 120 Waltham, MA, USA). The primer sequences were designed using Primer Express™ Software v3.0.1 (ThermoFisher Scientific, USA) (Supplemental material).

### *In Silico* Analysis

For biomarker discovery, we used the RNA-seq data of 549 uterine cancer patients extracted from TCGA, PanCancer Atlas using the publicly available cBioPortal online database https://www.cbioportal.org (21). The TCGA data contains 115 serous and 410 endometrioid endometrial cancers. Using the same program, the clinical and genomic profiles that include the gene expression levels, mutation profile, copy-number variance (CNV) as well as protein expression were assessed. In addition, the Kaplan–Meier survival analysis for different subgroups of endometrial cancer patients was also assessed according to cBioPortal instructions.

To investigate the association between different subgroups including obese and non-obese as well as LN negative and LN positive, the raw data files were extracted from the cBioPortal database and filtered for further analysis using our in-house pipeline for normalization and variant filtration. Height and weight were available for each patient and BMI was calculated using the following formula, BMI= weight (kg)/(height (m))^2^. The shortlisted DEGs were obtained thought selection of genes that showed 2-fold change between the different groups and adjusted p value <0.05 as cut-offs. According to those conditions, the endometrioid subgroup were subdivided according to their BMI into obese (BMI>30) and non-obese (BMI<30). Each group was further divided into those with no pelvic LN involvement (LN-) and those with any number of positive pelvic LN (LN+). The four groups were compared in terms of clinical, pathological, genomic and transcriptomic profiles. Differentially expressed genes between the different groups were shortlisted.

### Network and Pathway Interaction

Metscape online tool (http://metascape.org/) was used to investigate the network and interaction between the differential expressed genes and their biological function. This is a biological database and web resource of known and predicted protein–protein interactions. We used this tool to highlight the significance of the potential connectivity network of our genes that need to be considered for the full understanding of the biological process.

### Tissue Microarray

The tissue microarray (TMA) consists of 35 cases of endometrioid endometrial cancer (15 obese LN+, 20 obese LN-), and 10 normal adjacent tissue. Construction was performed as previously described by our group (22). Briefly, tissue cylinders with a diameter of 0.6 mm were punched from representative tumor areas of the tissue block selected for high tumor content, using a semiautomatic robotic precision instrument. Two sections of the TMA blocks were transferred to an adhesive coated slide system (Instrumedics Inc., Hackensack, New Jersey). Slides of the finished blocks were used for immunohistochemistry analysis.

### Immunohistochemistry (IHC) Analysis

IHC was performed at the Segal Cancer Centre Research Pathology Facility (JGH) using standard IHC protocols. Every tissue had a section stained with hematoxylin and eosin (H&E) and tumor content was assessed. Antibody immunostaining was performed using the Discovery XT Autostainer (Ventana Medical System) and a standardized diagnostic applications protocol. Two reference tissue samples were included on each slide as a positive and negative control. Sections were analyzed by conventional light microscopy.

CEMIP, RPL36A, CXCL14, EPHX3, TMEM254, CLIC6 and HMNC1 showed mainly cytoplasmic expression in the tumor epithelial cells. The immunoreactivity of all antibodies was classified according to the intensity into four categories. Cases with no evidence of cytoplasmic immunoreactivity considered as 0 score. Cases with weak immunoreactivity considered as +1 score. Cases with moderate immunostaining were given +2 score, +3 score were given to cases that showed strong immunoreactivity. For statistical analysis, cases considered positive if they are scored with +2, +3 category and negative if they fall into 0, + 1 categories. All the cases were reviewed by 2 pathologists that blindly and independently evaluated the slides.

### Statistical Analysis

The shortlisted DEGs were obtained thought selection of genes that showed 2-fold change between the different groups and adjusted p value <0.05 as cut-offs. All results are presented as the mean ± SEM for at least three independent experiments. The difference between groups was analyzed using Student’s *t*-test, and *P* < 0.05 was considered statistically significant.

## Results

### Categorization of Endometrioid Endometrial Cancer According to BMI to Investigate Top Differentially Expressed Genes (DEGs) That Can Differentiate Obese From Non-Obese Patients

**Figure 1:**
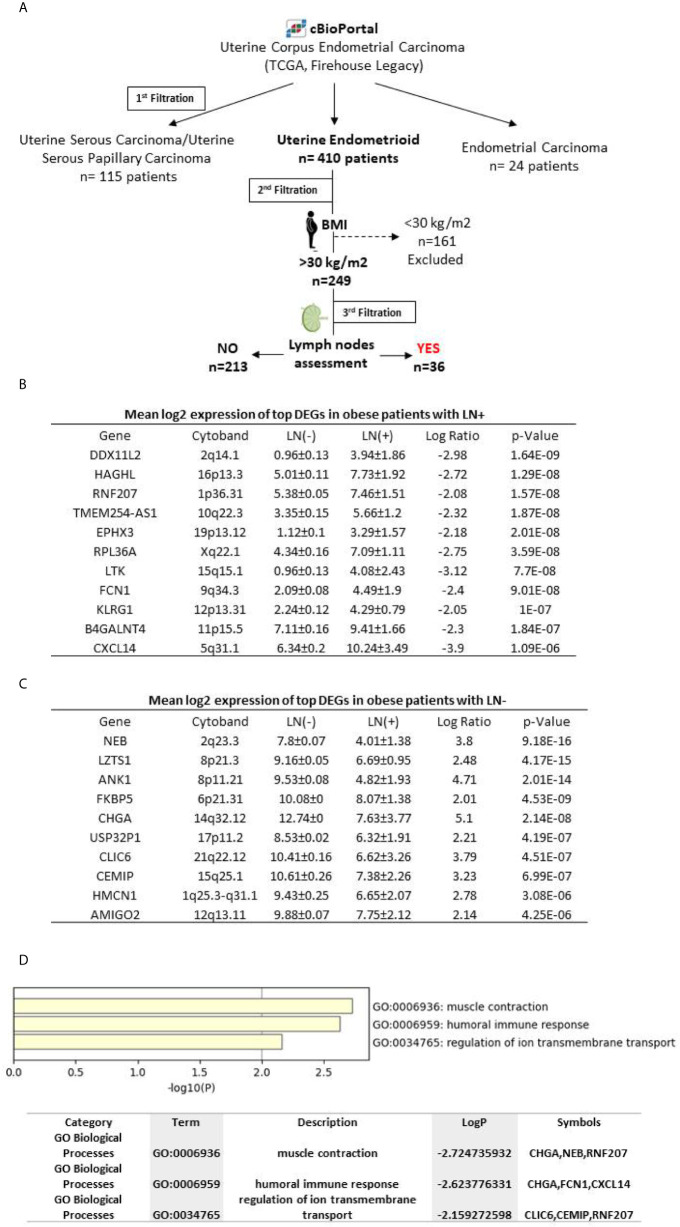
**(A)** Schematic workflow describing biomarker discovery, **(B)** Log2 expression levels of the differential expressed genes discovered in LN+ patients **(C)** Log2 expression levels of the differential expressed genes discovered in LN- patients, **(D)** Pathway analysis of differentially expressed genes using metscape tool for the retrieval of interacting genes. Enriched biological process and molecular functions of those proteins are included.

For biomarker discovery, we used the RNA-seq data of uterine cancer patients extracted from TCGA PanCancer Atlas using the publicly available cBioPortal online database. The RNA-seq data was extracted from 549 uterine cancer patients and filtrated to classify them according to cancer subtypes. Using the same program, the clinical and genomic profile that includes the gene expression levels, mutation profile, copy-number variance (CNV) was assessed. In addition, the Kaplan–Meier survival analysis for different subgroups of endometrial cancer patients was also assessed according to cBioPortal instructions. The patients were filtrated according to cancer subtypes, 115 serous, 410 endometrioid and 24 other carcinomas were obtained. The second filtration was performed in the cohort of endometrioid EC (410 patients) where the raw data files were extracted from the cBioPortal database and filtered accordingly for further analysis using our in-house pipeline for normalization and variant filtration. The DEGs were obtained through selection of genes that showed 2-fold change or high between the different groups and adjusted p-value<0.05 as cut-offs. Next, using the associated clinical information provided by the databases, we classified endometrioid EC patients in obese (BMI>30) (249 patients) and non-obese (BMI<30) (161 patients) according to their BMI, that was obtained from the patient’s height and weight, using the following formula, BMI = kg/m2. The obese group was further divided into those with no pelvic LN (LN-) (213 patients) and those with any number of pelvic LN (LN+) (36 patients) (**Figure 1A**).

### Identification of Top Differential Genes Between LN Negative and Positive Cases in Obese Endometrioid Cancer Patients

For better understanding of the association between obesity and LN involvement in endometrioid cancer subtype, we stratified patients with BMI >30 according to their LN status. The top DEGs between LN- and LN+ subgroups were identified. Eleven genes (CXCL14, FCN1, EPHX3, DDXIIL2, TMEM254, RNF207, LTK, HAGHL, RPL36A, BHGALNT4 and KLRC1) were up regulated in LN+ cases compared to LN- (**Figure 1B**). In contrast, a different set of eleven DEGs (NEB, ANK1, AMIGO2, LZTS1, FKBP5, CHGA, USP32PI, CLIC6, CEMIP and HMCN1) were found to be up regulated in LN- samples compared to LN+ (**Figure 1C**).

In order to investigate the network and interaction between the differentially expressed genes and their biological function, we used metscape online tool. Interestingly, we found that the panel of genes are directly involved in muscle contraction (CHGA, NEB and RNF207), humoral immune response (CHGA, FCN1 and CXCL14) and regulation of ion transmembrane transport (CLIC6, CEMIP and RNF207) as shown in **Figure 1D**.

### Validation of the LN Involvement Top DEGs in a Patients’ Cohort From Our Tissue Bank

**Figure 2:**
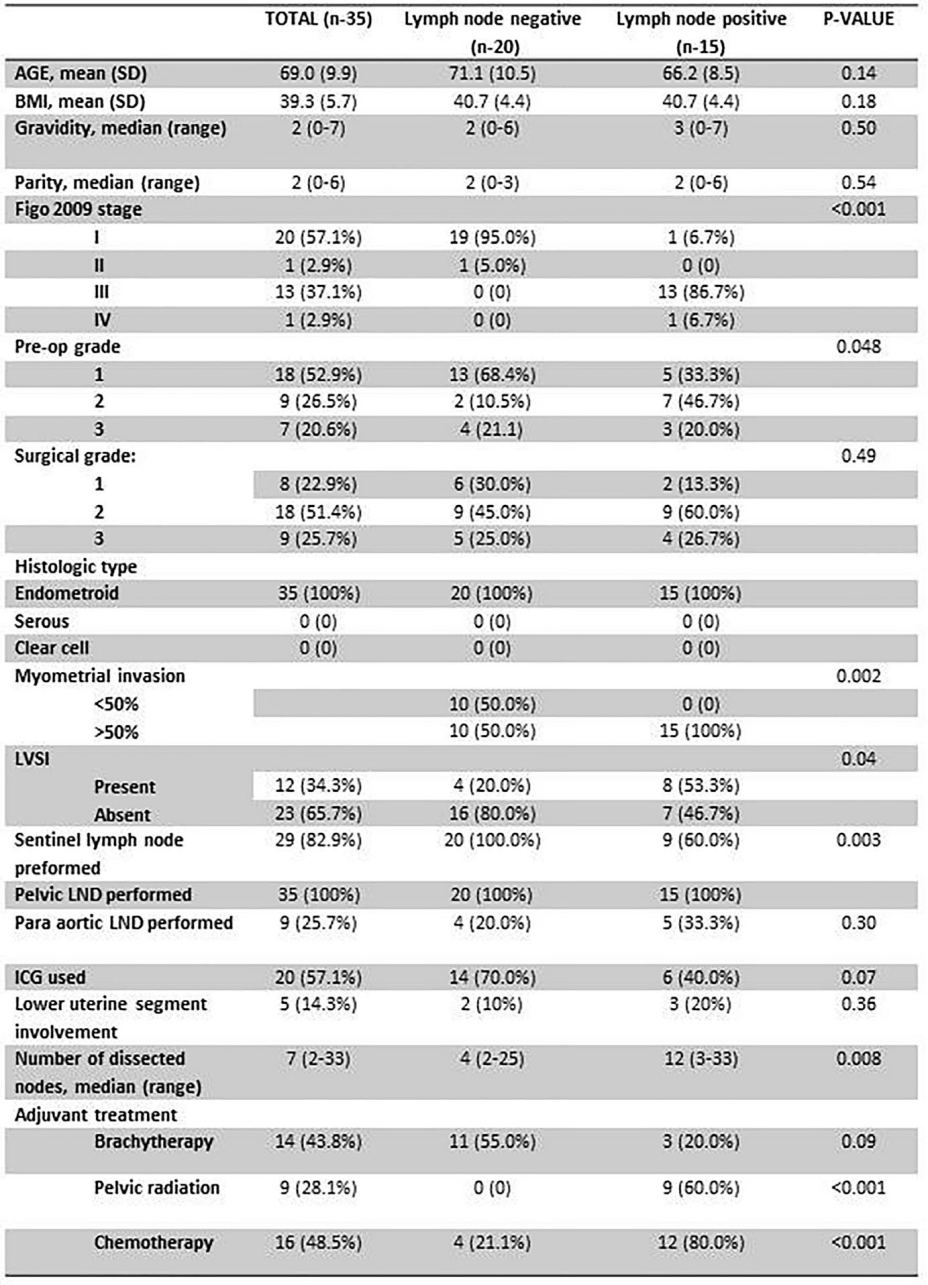
Basic characteristics of the validation cohort.

Thirty-five samples of endometroid EC patients were retrieved from our biobank and were used for validation. This panel includes LN+ (n=15) and LN- (n=20) patients. Overall median age was 69 years old, median BMI was 39.3kg and the majority of patients had a preoperative ASA score of 1 or 2 with no significant difference between the two groups. While all patients underwent LN dissection, 82% had SLN mapping performed (p=0.003). In the LN+ group deep myometrial invasion (>50%) was observed in all patients compared with LN- group (p=0.002) (**Figure 2**).

**Figure 3:**
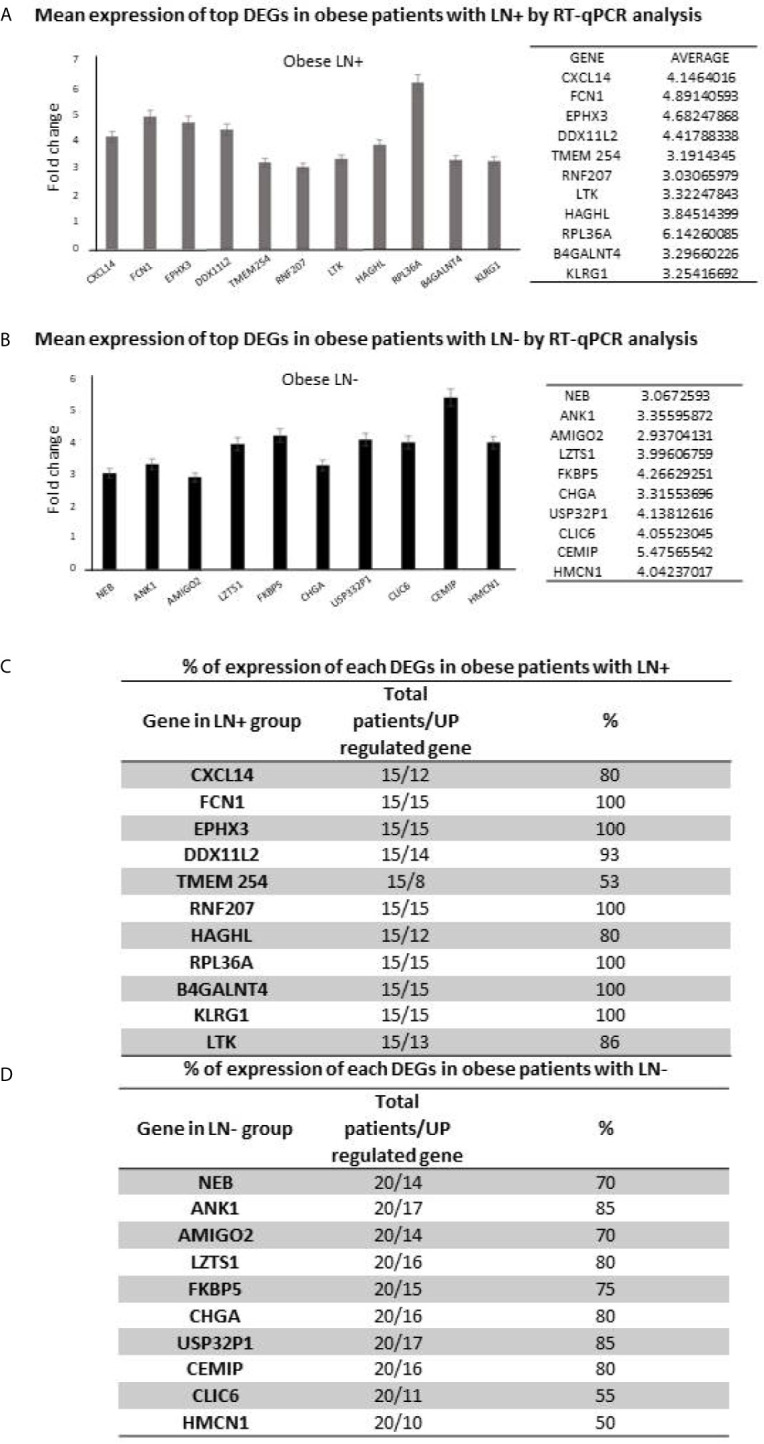
mRNA expression levels (fold change) of the differential expressed genes in **(A)** obese patients with LN+ and **(B)** LN- patients, using RT-qPCR analysis. **(C)** Percentage of expression of the differential expressed genes in patients with LN+ and **(D)** LN- patients.

**Figure 4:**
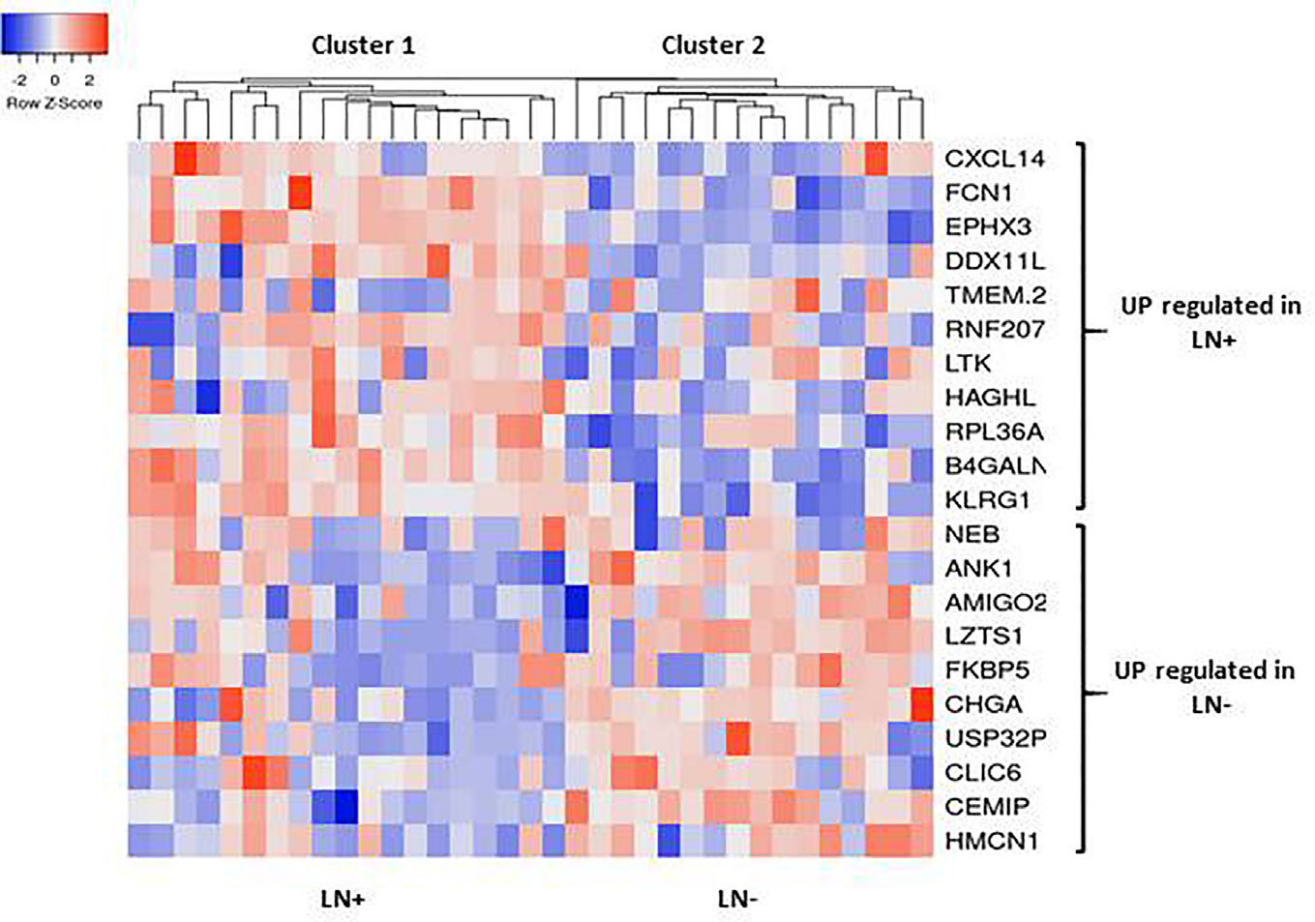
Hierarchical clustering heat map of differential expressed genes (rows) between LN+ and LN- tumors. Red indicates high expression and blue low expression.

Following the identification of the candidate genes, we next validated the in-silico approach using qPCR analysis in this patient cohort. Samples of 15 obese EC patients with LN involvement and 20 obese EC patients with negative nodes were assessed. As shown in **Figure 3A**, a panel of eleven genes were identified to be upregulated in the LN+ group (CXCL14, FCN1, EPHX3, DDXIIL2, TMEM254, RNF207, LTK, HAGHL, RPL36A, BHGALNT4 and KLRC1). Ten genes were found upregulated in ≥70% of the patients and TMEM254 gene was highly expressed in 53% of the patients. (**Figure 3C**). Moreover, in the LN- group out of ten identified genes that were upregulated, only 8 genes (NEB, ANK1, AMIGO2, LZTS1, FKBP5, CHGA and USP32P) were able to be validated in the majority of the patients (≥70% of expression), while CLIC6 and HMCN1 were found expressed in only 50% of the patients (**Figures 3B, D**). Most importantly, 10 out 11 genes in LN+ group are potential precise predictors of LN involvement (>80% expression) in obese woman with EC. Furthermore, hierarchical clustering of patients according to the expression of the genes showed that LN- patients were clustered together, while those in the LN+ group were clustered separately (**Figure 4**).

### CXCL14, EPHX3, TMEM 254, RPL36A, LZTS1, CLIC6, CEMIP and HMCN1 Protein Expression Levels Were Identified in the Validation Cohort Tissue Microarrays (TMA)

**Figure 5:**
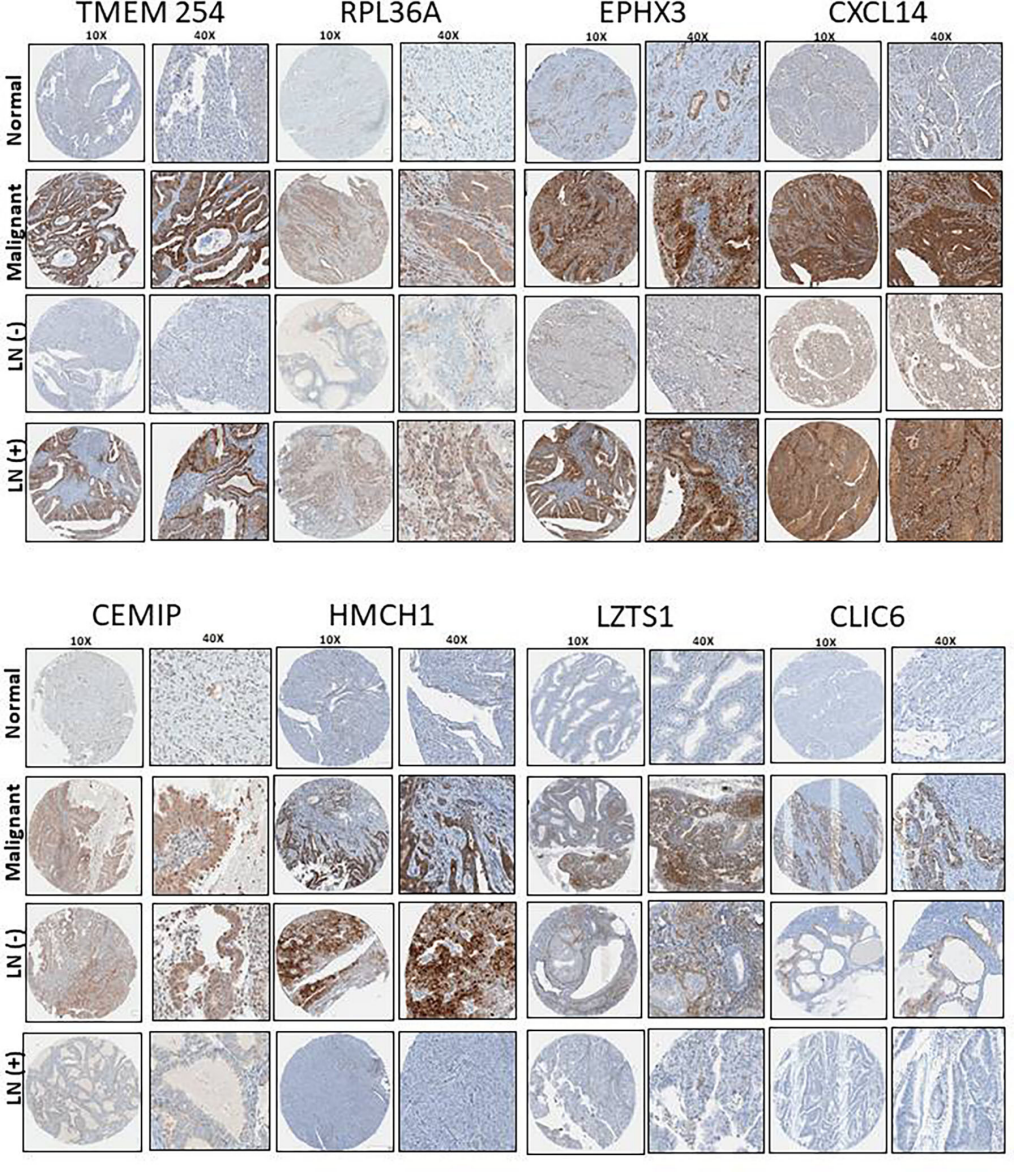
TMA representative images (10X and 40X) of the protein expression levels of the shortlisted genes and its association with LN status in our patient cohort consisting on 35 obese patients with EC.

Using the portal of the publicly available Human Protein Atlas of the TCGA, we were able to find the protein expression status of our identified genes in EC tissue. We found that eight genes from our panel were expressed at the protein level: CXCL14, EPHX3, TMEM254, RPL36A (LN+), LZTS1, CLIC6, CEMIP and HMCN1 (LN-). Next, using immunohistochemistry we examined the protein expression levels using TMAs containing the tissue samples of our validation cohort. Our analysis revealed that the protein expressions of all of the 8 genes were confirmed to be upregulated in the EC samples compared to the normal tissue (**Figure 5**).

Furthermore, LN+ genes where highly expressed at the protein level with statistical significance (CXCL14 (P=0.045), EPHX3 (P=0.023), TMEM254 (P=0.024) and RPL36A (P=0.050)). In contrast, the same trend was also observed in LN- patients but we did not observe any statistically significant difference (LZTS1 (P= 0.18), CLIC6 (P=0.81), CEMIP (P=0.35) and HMCN1 (P=0.14)) (**Table 1**).

**Table 1:** The expression levels of the 8 shortlisted genes and its association with LN status in our patient cohort consist of 35 obese patients diagnosed with endometrioid endometrial cancer.

Protein	IHC	NAT	Malignant	P-value	LN negative	LN positive	Total	P-value
**HMCN1**	Positive	3 (30%)	19 (54.28%)	0.31	13 (65%)	6 (40%)	19	0.14
	Negative	7 (70%)	16 (45.71%)		7 (35%)	9 (60%)	18	
**CLIC6**	Positive	0 (0%)	4 (11.42%)	0.62	3 (15%)	1 (7.1%)	4	0.81
	Negative	10 (100%)	31 (88.57%)		17 (85%)	14 (93.33%)	31	
**CEMIP**	Positive	5 (50%)	25 (71.42%)	0.37	16 (80%)	9 (60%)	25	0.35
	Negative	5 (50%)	10 (28.57%)		4 (20%)	6 (40%)	10	
**LZTS1**	Positive	4 (40%)	23 (65.71%)	0.27	15 (75%)	8 (53.33%)	23	0.18
	Negative	6 (60%)	12 (34.28%)		5 (25%)	7 (46.67%)	12	
**CXCL14**	Positive	1 (10%)	24 (70.5%)	0.001	11 (55%)	13 (86.67%)	24	0.045
	Negative	9 (90%)	11 (29.5%)		9 (40%)	2 (13.33%)	11	
**EPHX3**	Positive	4 (40%)	23 (65.71%)	0.14	10 (50%)	13 (86.67%)	23	0.023
	Negative	6 (60%)	12 (34.28%)		10 (50%)	2 (13.33%)	12	
**RPL36**	Positive	1 (10%)	19 (54.28%)	0.03	8 (40%)	11 (73.33%)	19	0.050
	Negative	9 (90%)	16 (45.71%)		12 (60%)	4 (26.67%)	16	
**TMEM254**	Positive	3 (30%)	18 (52.43%)	0.23	7 (35%)	11 (73.33%)	17	0.024
	Negative	7 (70%)	17 (48.57%)		13 (65%)	4 (26.67%)	18	

*IHC, Immunohistochemistry; NAT, normal adjacent tissue; LN, lymph node.

## Discussion

The association between EC and obesity has been extensively investigated and prior reports highlighted the critical role of obesity in the development of EC, impact on patient’s outcome and determination of therapeutical options (23). Indeed, surgical staging including LN assessment represents a cornerstone in the standard of care for EC (24, 25) however, in obese women, surgical intervention comprises significant challenges due to the patient’s comorbidities (cardiovascular, respiratory and metabolic diseases) and the peri- and post-operative risks (vascular injury and lymphedema) (12). Furthermore, our research group showed previously a positive correlation between morbid obesity and lower detection rate of SLNs (12, 18). This highlights the value of discovering novel markers that can predict LN involvement in the preoperative period. The implementation of such biomarkers that indicate the probability for LN involvement will be valuable not only to improve patient’s stratification before surgery, but also to predict patient’s prognosis.

The current study approach was based on using large publicly available databases to shortlist potential genes that can predict LN involvement. This was followed by two levels of validation through RT-qPCR and immunohistochemistry using in-house patient’s samples. Interestingly, we found a panel of eleven genes (CXCL14, FCN1, EPHX3, DDXIIL2, TMEM254, RNF207, LTK, HAGHL, RPL36A, BHGALNT4 and KLRC1) that were upregulated in patients with LN involvement. Among these genes, CXCL14, EPHX3, TMEM254, and RPL36A were able to detect LN involvement in obese women with EC at the mRNA and protein levels. Moreover, NEB, ANK1, AMIGO2, LZTS1, FKBP5, CHGA, USP32PI, CLIC6, CEMIP and HMCN1 genes were found to be down regulated.

In support of these findings, recently Chemokine (C-X-C Motif) Ligand 14 (CXCL14) was recently proposed through CXCL14-CXCR4 and CXCL12-CXCR4 axes to be essential for the EC invasion process as well as myometrial invasion (26). Similarly, the ribosomal protein L36A (RPL36A) was also shown to play a role in the proliferation of malignant cells and was proposed as a new target for anticancer therapies. Moreover, it was overexpressed and associated with cellular proliferation in metastatic hepatocellular carcinoma (27). In addition, RPL36A was found to be one of 11 alternative splicing genes signature that can predict the prognosis of EC and was proposed as a potential target for new therapies (28). The function of the Epoxide Hydrolase 3 (EPHX3, and also known as ABDH9) is not fully understood. However, its expression, in addition to other alpha-beta hydrolase, was found to be increased in some cancers like bladder, lung carcinoids and acute myeloid leukemia (29–31).

In contrast, Leucine zipper putative tumor suppressor 1 (LZTS1), that was found to be down regulated in obese EC patients with LN involvement, is known to be a tumor suppressor gene and its expression was confirmed to be reduced in a number of malignancies including breast, esophageal and prostate cancers. Similarly, Hemicentin 1 (HMCN1, also known as fibulin 6 (FBLN6)), is an extra cellular matrix (ECM) protein, that is believed to be essential for stable cell-to-cell interactions as well as stabilization to ECM structure (32). For that reason, it was proposed to function as a metastatic suppressor in gallbladder cancer, head and neck and breast cancers (33–35). Moreover, CLIC6, is a member of the Chloride Intracellular Channel (CLIC) family, was found to be modulated in many cancers and its expression was found to be correlated with favorable outcome and better survival (36).

One of the limitations of this study is the sample number of the validation cohort, for that reason we divided the cases into only LN positive and LN negative groups. Further studies are needed to investigate not only the presence or absence of LNs, also the size and number of the LN involved that can improve our understanding of the predictive value of these markers.

In future studies, the incorporation of these predictive markers with other novel molecular groups and clinicopathological prognostic factors, might be essential to improve patient’s stratification and risk assessment (37–42), that will tailor the management of endometrial cancer patients to avoid unnecessary surgical approaches. Moreover, while our results were focused on patients with endometrioid endometrial cancer further studies are needed to investigate these new biomarkers in other histological and molecular subgroups (43, 44). Notably, we can highlight that the validation of our genes using immunohistochemical analysis provide a reliable, cheaper, and widely available tool (45–47). For that reason, the accuracy of our panel at the immunohistochemistry level highlighted its feasibility and cost-effective alternative in the clinical setting.

To conclude, using publicly available microarray datasets in this study, we were able to discover a set of genes that was associated with LN status. Furthermore, the candidate genes were validated in an independent cohort from our biobank, using RT-qPCR and immunohistochemistry. Our results suggest that this novel panel might enable to predict LN involvement in obese EC patients preoperatively and may help to individualize the therapeutic approach and to reduce surgical morbidities in this high-risk population. Future studies are needed to evaluate the clinical application of these results and to investigate whether LN assessment, on the basis of this genetic panel, could be safely omitted in obese patients with EC.

## Data Availability Statement

Publicly available datasets were analyzed in this study. This data can be found here: https://www.cbioportal.org.

## Author Contributions

LL and VL-O performed RNA extractions, qPCRs and analyzed the results. MH carried out all the in-silica analysis. IH performed the histopathological evaluation. CM and EM helped in the analysis of the clinical information. VL-O conceptualization, research design and wrote the manuscript. LK, SL, and SS revised and edited the manuscript. AY and WG research design, supervised the study and drafting the manuscript. All authors made substantial contributions to the conception, design, acquisition of data, analysis and interpretation of data, all authors have been involved in revising and critically evaluating the manuscript for important intellectual content. In addition, each author has agreed to be accountable for the accuracy and integrity of this research work. All authors contributed to the article and approved the submitted version.

## Funding

This work was supported by grants from the Israel Cancer Research Fund (ICRF), the Gloria’s Girls Fund, and the Susan and Jonathan Wener Fund.

## Conflict of Interest

The authors declare that the research was conducted in the absence of any commercial or financial relationships that could be construed as a potential conflict of interest.

## Supplementary Material

Supplementary Figure 1 Fold change expression of differential expressed genes in each obese patient with **(A)** LN+ and **(B)** LN-.
