# Supplementary figures and images for: Identification of Predictive Biomarkers for Lymph Node Involvement in Obese Women With Endometrial Cancer
Source: Front Oncol. 2021 Jul 7;11:695404. doi: 10.3389/fonc.2021.695404 (PMC8292832; doi:10.3389/fonc.2021.695404)

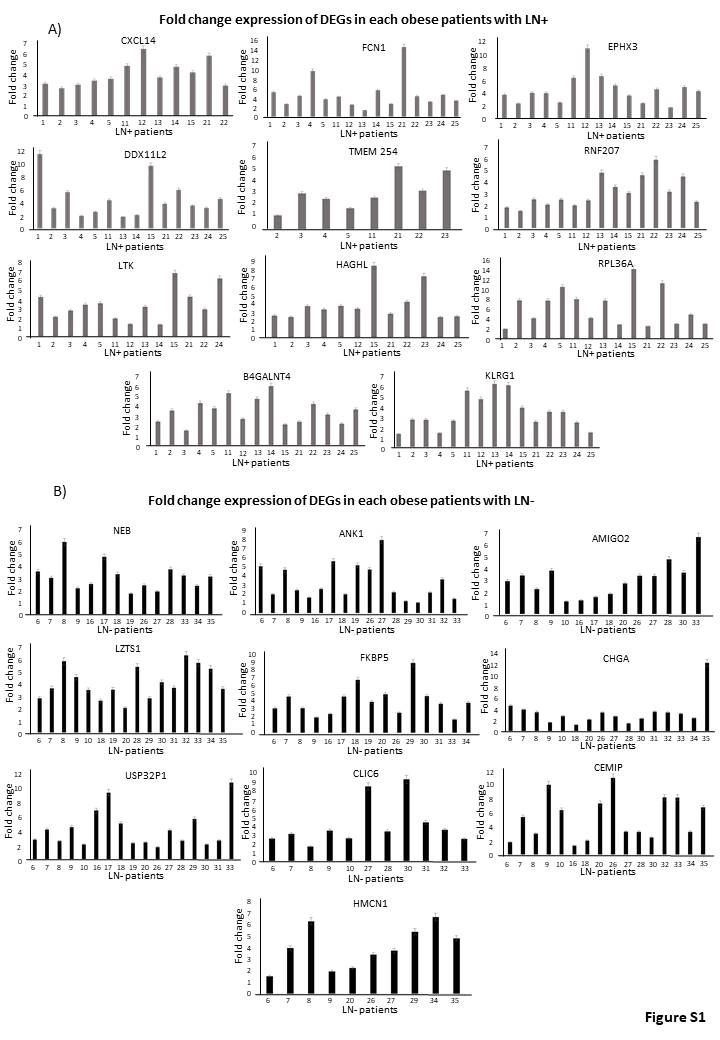

Supplement: Supplementary Figure 1 — Fold change expression of differential expressed genes in each obese patient with (A) LN+ and (B) LN-. [file Image_1.jpg]
